# Multi-Pharmacology of Berberine in Atherosclerosis and Metabolic Diseases: Potential Contribution of Gut Microbiota

**DOI:** 10.3389/fphar.2021.709629

**Published:** 2021-07-09

**Authors:** Shengjie Yang, Dan Li, Zongliang Yu, Yujuan Li, Min Wu

**Affiliations:** ^1^ Guang’an men Hospital, China Academy of Chinese Medical Sciences, Beijing, China; ^2^ Xiyuan Hospital, China Academy of Chinese Medical Sciences, Beijing, China

**Keywords:** berberine, atherosclerosis, metabolic diseases, gut microbiota, inflammation, metabolites

## Abstract

Atherosclerosis (AS), especially atherosclerotic cardiovascular diseases (ASCVDs), and metabolic diseases (such as diabetes, obesity, dyslipidemia, and nonalcoholic fatty liver disease) are major public health issues worldwide that seriously threaten human health. Exploring effective natural product-based drugs is a promising strategy for the treatment of AS and metabolic diseases. Berberine (BBR), an important isoquinoline alkaloid found in various medicinal plants, has been shown to have multiple pharmacological effects and therapeutic applications. In view of its low bioavailability, increasing evidence indicates that the gut microbiota may serve as a target for the multifunctional effects of BBR. Under the pathological conditions of AS and metabolic diseases, BBR improves intestinal barrier function and reduces inflammation induced by gut microbiota-derived lipopolysaccharide (LPS). Moreover, BBR reverses or induces structural and compositional alterations in the gut microbiota and regulates gut microbe-dependent metabolites as well as related downstream pathways; this improves glucose and lipid metabolism and energy homeostasis. These findings at least partly explain the effect of BBR on AS and metabolic diseases. In this review, we elaborate on the research progress of BBR and its mechanisms of action in the treatment of AS and metabolic diseases from the perspective of gut microbiota, to reveal the potential contribution of gut microbiota to the multifunctional biological effects of BBR.

## Introduction

Atherosclerosis (AS), atherosclerotic cardiovascular diseases (ASCVDs), and metabolic diseases (including diabetes, obesity, dyslipidemia, and nonalcoholic fatty liver disease (NAFLD)) are serious hazards to human health (O’Neill and O’Driscoll, 2015; Herrington et al., 2016). Because these diseases result from the combined effects of various pathological factors, it is difficult to fully clarify their pathogenesis. Currently, western medicine and lifestyle interventions are the mainstream treatments for these diseases (Kong et al., 2020). For example, statins, hypoglycemic drugs, and antithrombotic drugs are widely used in clinical practice (Law and Rudnicka, 2006; Chatterjee et al., 2017; Squizzato et al., 2017). However, adverse effects such as gastrointestinal discomfort, myalgia, liver dysfunction, and cardiovascular lesions (Adhyaru and Jacobson, 2018; Wang and Hoyte, 2019) limit the use of these drugs. In recent years, effective drugs for the treatment of AS and metabolic diseases based on natural products have attracted increasing attention from researchers (Xu et al., 2018; Wang et al., 2019). Berberine (BBR) is an important isoquinoline alkaloid found in several medicinal plants, including *Coptis chinensis* Franch, cortex phellodendri, and *Berberis asiatica* (Xu et al., 2021). The pharmacological effects of BBR have been extensively studied, including its anti-inflammatory (Takahara et al., 2019; Zeng et al., 2021), anti-obesity (Ilyas et al., 2020), antidiabetic (Zhang et al., 2010; Wang et al., 2011a), anti-atherosclerotic (Ke et al., 2020; Tan et al., 2020), antilipidemic (Ju et al., 2018; Ren et al., 2020), and cardioprotective effects (Yu et al., 2018). In view of the poor oral bioavailability of BBR and its extremely low maximum plasma concentration, there is increasing evidence indicating that the gut microbiota is a crucial mediator that regulates the pharmacokinetic and biological effects of BBR (Chen et al., 2011; Wang et al., 2017a; Habtemariam, 2020a). Studies over the past decade have also demonstrated that the gut microbiota is closely related to AS and metabolic diseases (Barrington and Lusis, 2017; Fan and Pedersen, 2021). Therefore, targeting the regulation of gut microbiota structure, gut microbiota-dependent metabolites, and related downstream pathways may be a novel tractable therapeutic strategy for the treatment of AS and metabolic diseases. In this context, the purpose of this review is to elaborate on the research progress of BBR and its mechanisms of action in the treatment of AS and metabolic diseases from the perspective of gut microbiota, to reveal the potential contribution of the gut microbiota to the multifunctional biological effects of BBR.

## Berberine Bioavailability and Gut Microbiota

Although studies have reported that BBR has good therapeutic effects against a variety of diseases, including AS and metabolic diseases, its structural characteristics result in poor water solubility and intestinal malabsorption. Evidence from studies in rats suggests that the absolute bioavailability of BBR after oral administration is far less than 1% (Chen et al., 2011; Wang et al., 2017a). Current studies have reported that p-glycoprotein inhibitors (Chen et al., 2011), different BBR-based functional nanocarriers (Pang et al., 2015; Li et al., 2020a; Ma et al., 2020) and other physicochemical modification approaches (Habtemariam, 2020b) can increase the bioavailability of BBR, leading to enhanced anti-AS effects and enhanced diabetes and inflammatory disease treatment efficacy. Notably, recent studies have shown that one of the connections between the expected poor oral bioavailability of BBR and its pharmacological effects is the gut microbiota. BBR can be converted by the gut microbiota to dihydroberberine (dhBBR), an intestine-absorbable form with a 5-fold higher absorption rate than BBR in animals. dhBBR can then be re-oxidized to BBR after being absorbed in intestinal tissues and entering the blood (Feng et al., 2015). Li et al. (2020b) evaluated the effects of BBR and its structural analogs on different individual gut microbiota cultures *in vitro*. The results showed that dhBBR had a similar effect to that of BBR in enriching the abundance of *Akkermansia,* and dhBBR displayed fewer overall adverse effects on gut microbiome function, such as sustaining butyrate levels.

Notably, the differential pharmacokinetics of BBR may be related to alterations in gut microbiota in healthy and pathological states. Nitroreductases (NRs), which are bacterial enzymes, have been shown to be crucial factors in promoting the intestinal absorption of BBR. A study by Wang et al. showed that the proportion and activity of NR-producing bacteria were increased in hamsters fed a high-fat diet (HFD), and the corresponding bioavailability of BBR was higher than that of hamsters fed a normal diet. The serum BBR levels in patients with hyperlipidemia were also higher than those in healthy individuals after oral administration (Wang et al., 2017b). Similarly, Feng et al. described the gut microbiota-regulated pharmacokinetics of orally administered BBR in beagle dogs. These researchers revealed that the abundance of both butyrate- and NR-producing bacteria was increased in dogs fed BBR (50 mg/kg/d) for 7 days (Feng et al., 2018). Under the pathological condition of diabetes, the absorption and metabolism abilities of BBR were also different from the normal state, and the serum BBR levels in pseudo-sterile diabetic rats were markedly lower than those in diabetic rats, suggesting an important role of the gut microbiota (Du et al., 2020). In addition, differences in gut microbiota among individuals and ethnicities may contribute to the pharmacokinetic differences in BBR. Alioga et al. investigated the effect of gut microbial variations and metabolic capacity on BBR pharmacokinetics in healthy African and Chinese males. Their findings revealed that the abundance of *Prevotella*, *Bacteroides*, and *Megamonas* in Chinese was more enriched. More extensive metabolism and higher metabolites were also found, which might be part of the reason for the significant differences in BBR pharmacokinetics between the two groups (Alolga et al., 2016). The biological effects of BBR on gut microbiota may also be influenced by the dose. In our previous study, we observed the effects of different doses of BBR on AS and gut microbiota regulation in HFD-fed apolipoprotein E (ApoE)^−/−^ mice, and found that the anti-AS effect of high-dose BBR (i.g. 100 mg/kg/d) was superior to that of low-dose BBR (i.g. 50 mg/kg/d) (Wu et al., 2020). Most doses in the relevant studies described in this review were 50, 100, or 200 mg/kg, as shown in Table 1. It should be noted that most studies were conducted in animal models, and only a few high-quality studies involved humans and changes in human microbiota. Therefore, the published data in this section are incomplete and need to be resolved in future studies.

**TABLE 1:** BBR-mediated structural and compositional alterations in the gut microbiota in AS and metabolic diseases.

Pathological condition	Subject or model	Interventions/Dosage	Outcome	Reference
AS	ApoE^−/−^ mice fed with HFD	BBR; administered to drinking water (0.5 g/L) for 14 weeks	*Akkermansia spp*. ↑; proinflammatory cytokines ↓; gut barrier integrity ↑	Zhu et al. (2018a)
AS	Male ApoE^−/−^ mice fed with HFD	BBR; i.g. 50 mg/kg twice weekly for 12 weeks	Firmicutes and Verrucomicrobia ↑; hepatic FMO3 expression ↓; serum TMAO levels ↓	Shi et al. (2018)
AS	ApoE^−/−^ mice fed with HFD	BBR; i.g. 50 and 100 mg/kg for 13 weeks	*Alistipes*, *Allobaculum*, *Blautia*, *Roseburia*, *Turicibacter* ↑; serum lipid and systemic inflammation levels ↓; potential for TMAO production ↓	Wu et al. (2020)
AS	Choline-fed ApoE KO mice	BBR; 100 or 200 mg/kg for 4 months	*Bacteroides*, *Prevotella*, *Parabacteroides*, *Alloprevotella* ↑; TMA/TMAO production ↓; cutC and cntA ↓	Li et al. (2021)
Obesity and IR	HFD-fed rats	BBR, p.o. 100 mg/kg, for 8 weeks	SCFA-producing bacteria (*Blautia* and *Allobaculum*) ↑; fecal SCFA concentrations ↑; gut microbiota diversity ↓	Zhang et al. (2012)
Obesity	Diet-induced obese mice (C57BL/6 mice fed with HFD)	BBR; dietary supplementation at 100 mg/kg/day for 8 weeks	Ratio of F/B ↓	Sun et al. (2018)
Obesity	HFD-fed C57BL/6J mice	Rhizoma coptidis and BBR, p.o. 200 mg/kg, for 6 weeks	Both have similar effects: the fecal levels of Firmicutes and Bacteroidetes ↓; gut bacteria growth ↓; growth of *Lactobacillus in vitro* trials ↓	Xie et al. (2011)
Obesity and IR	HFD-fed obese rats	BBR, p.o. 200 mg/kg for 8 weeks	Protective bacteria like *Bifidobacterium* ↑; negative bacteria like *Escherichia coli* ↓; TG, LDL-C, FBG, and IR ↓; LPS-induced TLR4/TNF-α activation ↓	Liu et al. (2018)
Obesity	HFD-induced obesity in rats	BBR 100 or 200 mg/kg, metformin 200 mg/kg, i.g. for 8 weeks	Both have similar effects: SCFA-producing bacteria including *Allobaculum*, *Blautia*, *Bacteroides*, *Butyricicoccus*, and *Phascolarctobacterium* ↑	Zhang et al. (2015)
Obesity	HFD-induced obese rats	BBR; orally administrated with 150 mg/kg/day for 4 months	B/F ratio ↑; SCFA-producing bacteria Bacteroidetes, *Bilophila* ↑; genera from phylum Firmicutes (*Dorea*, *Roseburia*, and *Blautia*) ↓; genera from phylum Proteobacteria: *Sutterella*, *Desulfovibrio* ↑; GLP-1, NPY ↑; orexin A ↓	Sun et al. (2016)
Obesity	HFD-fed obese rats	BBR, p.o. 150 mg/kg, for 6 weeks	Restoring the gut barrier, reducing LPS levels, systemic inflammation and metabolic endotoxemia through modulating gut microbiota (gut microbiota diversity ↓; *Fusobacteria* and *Proteobacteria* ↑; *Firmicutes* and *Actinobacteria* ↓)	Xu et al. (2017)
Normal individuals	Grass carp (*Ctenopharyngodon idella*)	BBR, diet supplementation at 30 mg/kg	Serum glucose, TC, and TG levels ↓; regulating gut microbiota structure: F/B ratio ↓; enriched OTUs changed from mainly belonging to Firmicutes to Proteobacteria, Planctomycetes, Bacteroidetes, and Firmicutes	Pan et al. (2019)
T2D	Streptozotocin and high-fat/sucrose diet-induced diabetic rats	BBR, i.g. 500 mg/kg for 4 weeks	*Alloprevotella*, Bacteroidetes*,* Clostridia*,* Lactobacillales, and Prevotellaceae ↑; Bacteroidales, *Desulfovibrio*, Lachnospiraceae, and Rikenellaceae ↓; inflammation ↓; oxidative stress-related proteins (PI3K, GLUT2) ↑	Cui et al. (2018)
T2D	Diabetic Goto-Kakizaki rats	BBR, i.g. 200 mg/kg/d, for 8 weeks	*Muribaculaceae* ↓; Allobaculum ↑; Bacteroidetes ↓; B/F ratio ↓; improving metabolic parameters (weight, FBG, GLP-1, and homeostatic model assessment-IR)	Zhao et al. (2021)
Prediabetes or T2D	Zucker diabetic fatty rats	BBR, i.g. 100 mg/kg/d, for 3 weeks	Improved the gut microbiota structure and species diversity; food intake, FBG, IR, and plasma LPS levels ↓, *GLP-2* ↑; slow progression of prediabetes to T2D	Wang et al. (2021a)
T2D	Diabetic rat model	Gegen Qinlian Decoction and BBR	Both have similar effects: changed the overall structure of gut microbiota; butyrate-producing bacteria (*Faecalibacterium* and *Roseburia*) ↑; SCFAs levels in feces ↑; intestinal inflammation and blood glucose ↓	Xu et al. (2020)
T2D	db/db mice	BBR or metformin, i.g. 136.5 mg/kg for 11 weeks	Both have similar hypoglycemic effects; SCFA-producing bacteria (*Butyricimonas*, *Coprococcus*, *Ruminococcus*) ↑; probiotics (*Lactobacillus*, *Akkermansia*) ↑; *Prevotella*, *Proteus* ↓; intestinal SCFA content ↑	Zhang et al. (2019)
Obesity and IR	HFD-fed mice	BBR, p.o. 200 mg/kg, for 10 weeks	BCAA-producing bacteria (e.g., Clostridiales, Streptococcaceae, Clostridiaceae, Prevotellaceae, *Streptococcus*, and *Prevotella*) ↓	Yue et al. (2019)
T2D	Streptozotocin-induced diabetic rats	BBR, i.g. 200 mg/kg/day for 6 weeks	Richness and diversity of gut microbiota ↑; Bacteroidetes and *Lactobacillaceae* ↑; *Proteobacteria* and *Verrucomicrobia* ↓; concentration of AAAs in the colon and serum ↓	Yao et al. (2020)
T2D	db/db mice	BBR compounds (BBR, oryzanol, and vitamin B6)	Bacteroidaceae and Clostridiaceae ↑; microbiota-mediated DCA production ↑; TGR5 expression and GLP secretion ↑	Li et al. (2020c)
T2D	á	BBR (0.6 g per 6 pills, twice daily) or probiotics (4 g, once daily) +BBR, for 12 weeks	Hypoglycemic effect of BBR was mediated by the suppression of DCA biotransformation by *Ruminococcus bromii*	Wang et al. (2017d)
Prediabetes or T2D	300 newly diagnosed patients	*Bifidobacterium* and BBR, 16-weeks	Results have yet to be reported	Ming et al. (2018)
Dyslipidemia	HFD-induced hyperlipidemia in rats	BBR compounds, 150 mg/kg, p.o. for 4 weeks	Beneficial bacteria (*Bacteroides*, *Blautia*) ↑; *Escherichia* ↓	Li et al. (2016)
Dyslipidemia	HFD-induced hyperlipidemia in hamster	BBR, i.g. 100 mg/kg, for 2 weeks	Modulating the gut microbiota, F/B ratio ↑; transformation of CA into DCA ↓; intestinal bas elimination ↓	Gu et al. (2015)
Dyslipidemia	Hyperlipidemia hamsters	BBR, oral, 200 mg/kg, for 2 weeks	Blood butyrate levels ↑; BBR metabolites (M1, M2, and M3) ↑; production of SCFAs in gut microbiota ↑	Ma et al. (2019)
Normal condition	Several animal systems (SD rats, hamsters, ob/ob mice)	BBR, 100 mg/kg/day, oral	Abundance of butyrate-producing bacteria ↑; bacterial ATP production and NADH level ↓; butyrate levels ↑	Wang et al. (2017d)
Normal condition	Male C57BL/6 wild-type mice; isolated mouse cecal bacteria	BBR, *in vitro* (0.1, 1, and 10 mg/ml) for 4 h; *in vivo*-100 mg/kg for 5 days	*Clostridium cluster xiva* and *IV* ↓; BSH activity ↓; conjugated bas, especially TCA in the intestine ↑; intestinal FXR activation ↑	Tian et al. (2019)
Normal condition	Male C57BL/6 mice	Six doses of BBR (0, 3, 10, 30, 100, 300 mg/kg) i.g. for 2 weeks	*Bacteroides* in the terminal ileum and large bowel ↑; expression of Cyp7a1, Cyp8b1, and Ntcp ↑; BA production ↑	Guo et al. (2016)
Normal condition	Germfree mice colonized with gut bacterial consortium that is capable of functional BA metabolism	BBR, 100 mg/kg, oral gavage, for 27 days	Significant alterations in network topology of gut microbiota; cecal BA concentrations and excretion into the gastrointestinal tract ↑	Wolf et al. (2021)
NAFLD	HFD-induced NAFLD in rats	BBR, i.g. 150 mg/kg/d, for 4 weeks	Hepatic fatty degeneration ↓; occludin level ↑; improved intestinal barrier dysfunction; *Faecalibacterium prausnitzii* ↓and *Bacteroides* ↑	Li et al. (2017a)
NAFLD	HFD-induced NAFLD rats	BBR, i.g. 150 mg/kg/d, for 4 weeks	Protect gut barrier function; *Bacteroides* ↑; *Escherichia coli* and *Faecalibacterium prausnitzii* ↓	Li et al. (2020e)

AS, atherosclerosis; HFD, high-fat diet; TMAO, trimethylamine N-oxide; FMO3, flavin-containing monooxygenase-3; SCFA, short-chain fatty acid; T2DM, type 2 diabetes mellitus; FBG, fasting blood glucose; TC, total cholesterol; TG, triglyceride; LDL-C, low-density lipoprotein-cholesterol; IR, insulin resistance; BCAA, branched-chain amino acids; LPS, lipopolysaccharide; AAAs, aromatic amino acids; CA, cholic acid; DCA, deoxycholic acid; BA, bile acid; GLP, glucagon-like peptide; BSH, bile salt hydrolase; TCA, taurocholic acid; NAFLD, nonalcoholic fatty liver disease.

In conclusion, gut microbiota has received increasing attention as one possible connection between poor oral bioavailability of BBR and its pharmacological effects. Differential pharmacokinetics of BBR may be related to changes in gut microbiota under healthy and pathological states, differences in gut microbiota among individuals and ethnicities, as well as alterations in the gut microbiota of different BBR doses. More studies are expected to provide more detailed information on gut microbiota and BBR bioavailability.

## Berberine Improves Intestinal Barrier Dysfunction and Reduces Inflammation in Atherosclerosis and Metabolic Diseases

Many pathological conditions can be attributed to chronic inflammation, which is a well-known effector in AS and an indicator of increased cardiovascular risk. Obesity and other metabolic disorders can provoke and trigger inflammation and are risk factors for AS. In these diseases, intestinal barrier dysfunction has been shown to commonly occur and is closely related to systemic inflammation. Normal intestinal barrier function depends on the combined action of the mucus barrier between the intestinal epithelium and luminal contents and the apical junction complex composed of tight junctions, desmosomes, and adhesive junctions to regulate the permeability of epithelial cells (Odenwald and Turner, 2017; Lewis and Taylor, 2020). Under the pathological conditions of AS and metabolic diseases, increased inflammation, decreased mucus thickness, and decreased expression and function of epithelial and endothelial tight junction proteins lead to increased intestinal permeability. This in turn results in the displacement of gut bacteria and antigens into circulation and the activation of systemic inflammatory pathways, such as toll-like receptors (TLRs) and inflammasomes (Ma and Li, 2018; Feng et al., 2019). Serum lipopolysaccharide (LPS), a gut microbiota-derived metabolite, is the most commonly used marker for this process, and its increase can indicate the translocation of bacteria from the gut to circulation (Jonsson and Bäckhed, 2017).

Elevated levels of circulating LPS and inflammatory cytokines are thought to be relevant to atherosclerotic plaque formation and rupture (Libby et al., 2002; Hansson, 2005). When gut microbiota-derived LPS crosses the intestinal barrier and enters the bloodstream, it binds to toll-like receptors and initiates the innate proinflammatory response of the host, leading to endothelial dysfunction and the progression and vulnerability of atherosclerotic plaques (Beutler et al., 2003; Lu et al., 2012). The beneficial effects of BBR were demonstrated in a mouse model of AS induced by HFD, a diet that has been shown to increase plasma LPS levels by modulating the composition of gut microbiota, causing intestinal barrier dysfunction and enhancing intestinal permeability (Cani et al., 2008; Cani et al., 2009). In HFD-fed mice, BBR significantly reduced proinflammatory cytokine (including tumor necrosis factor (TNF)-α, interleukin (IL)-1β, and IL-6) and chemokine levels in the arteries and intestines, and alleviated HFD-induced metabolic endotoxemia, which was defined as insulin resistance (IR), impaired glucose tolerance (IGT), and obesity promoted by the LPS-induced systemic low-grade inflammatory state (Cani et al., 2007; Cani et al., 2008; Shi et al., 2018; Zhu et al., 2018a). Moreover, BBR administration increased colonic mucinous layer thickness and intestinal tight junction protein expression, both of which are associated with the restoration of intestinal barrier integrity. This beneficial effect of BBR was associated with the regulation of gut microbiota, particularly with increased abundance of *Akkermansia* (Zhu et al., 2018a), whose probiotic effects have been reported to include metabolic regulation, immune modulation, and protection of intestinal health (Plovier et al., 2017; Zhai et al., 2019).

BBR also improves intestinal mucosal barrier alteration, dysfunction, and inflammation in obesity, diabetes, and other metabolic diseases. A previous study showed that proinflammatory intestinal changes occurred in diabetic rats with 2.77-fold increased intestinal permeability. However, these changes were notably reversed by BBR treatment, and the anti-inflammatory mechanism may be related to the toll-like receptor 4 (TLR4)/myeloid differentiation factor 88 (MyD88)/nuclear factor (NF)-κB signaling pathway (Gong et al., 2017). Another study in rats with type 2 diabetes (T2D) induced by HFD and low-dose streptozotocin also supports the supposition that BBR treatment significantly restores damaged intestinal mucosal structure and intestinal permeability; the reduction in tight junction protein, zonula occludens 1 (ZO1), caused by the disease was also reversed. It was also observed that BBR reduced inflammatory cell infiltration, decreased plasma LPS levels, and improved endotoxemia (Shan et al., 2013). Notably, the alteration in gut microbiota composition correlated with the above effects of BBR, as detailed in Table 1. Studies by Zhang et al. (2019) showed that both BBR and metformin could regulate gut microbial composition, reduce serum LPS levels and intestinal inflammation, and reverse the decreased expression of tight junction proteins (ZO1 and occludin), thereby repairing the intestinal barrier structure in db/db mice. In obese mice, BBR-induced alterations in the gut microbiota led to notable reductions in plasma bacterial LPS levels, metabolic endotoxemia, and systemic inflammation. Suppression of the inflammatory response was related to decreased intestinal permeability and increased tight junction protein expression, which indicates the restoration of the intestinal barrier (Xu et al., 2017). In addition, BBR was found to have a significant protective effect on NAFLD by altering the gut microbiota and ameliorating intestinal barrier function (Li et al., 2017a).

**FIGURE 1:**
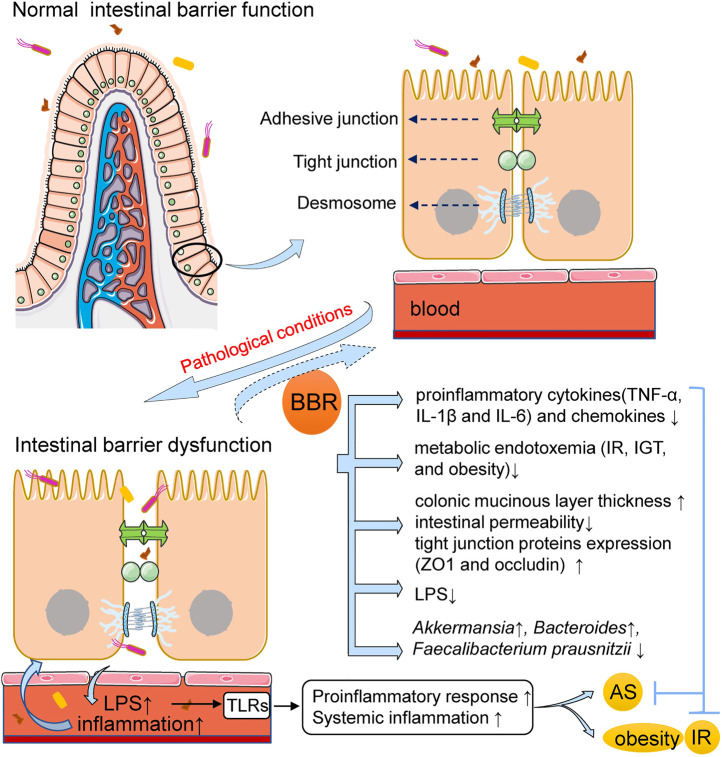
BBR improves intestinal barrier dysfunction and reduces inflammation in AS and metabolic diseases. Under the pathological conditions of AS and metabolic diseases, the intestinal permeability is increased, resulting in the displacement of gut bacteria and its-derived LPS into circulation and the activation of systemic inflammatory pathways. BBR administration can improve intestinal barrier dysfunction and reduce inflammation. The mechanism may involve that BBR increases colonic mucinous layer thickness and intestinal tight junction protein expression, both of which are associated with the restoration of intestinal barrier integrity; BBR reduces the expression of pro-inflammatory cytokines and chemokines, decreases plasma LPS levels and improves endotoxemia. The alteration in gut microbiota composition is correlated with the above effects of BBR. AS, atherosclerosis; BBR, berberine; IR, insulin resistance; IGT, impaired glucose tolerance; LPS, lipopolysaccharide; ZO1, zonula occludens 1; TLRs, toll-like receptors; TNF-α, tumor necrosis factor-α; IL-1β, interleukin-1β.

In conclusion, enhanced intestinal permeability has been shown to occur in patients with ASCVDs and metabolic diseases and in representative animal models. This increased intestinal permeability promotes the enhancement of systemic inflammation and changes in intestinal immune function, and can predict the risk of associated adverse cardiovascular events (Lewis and Taylor, 2020). Moreover, targeting the metabolic and immune functions of microbiota using natural herbal ingredients has been widely investigated as a possible therapy (Anlu et al., 2019). As a natural product, BBR can improve intestinal barrier dysfunction and reduce systemic inflammation caused by gut microbiota (Figure 1), thereby representing a promising treatment for AS, obesity, diabetes, and other metabolic diseases that trigger and provoke inflammation.

## Berberine Pharmacology and Gut Microbiota in Atherosclerosis

The mechanism of BBR in AS treatment has been under constant discussion, and the role that gut microbiota plays has become increasingly prominent. The rapid development and use of novel genome sequencing technologies have improved our understanding of the role of the human gut microbiota in AS, especially the alteration in gut microbiota composition and identification of the numerous metabolites produced in the intestines that are related to AS (Kanitsoraphan et al., 2018). Among them, the regulation of gut microbe-dependent trimethylamine N-oxide (TMAO) and short-chain fatty acids (SCFAs) appears to be particularly important for the anti-atherosclerotic effect of BBR.

### Anti-Atherosclerotic Effects of Berberine Through the Regulation of Gut Microbiota

Improvements in sequencing techniques and bioinformatics analysis of bacterial 16S rRNA genes have provided detailed information about gut microbiota changes in AS. An important effect of BBR related to its connection with AS is that it can regulate the composition of the gut microbiota. Zhu et al. (2018a) evaluated the association between the anti-atherosclerotic effect of BBR and alterations in specific bacterial taxa of the gut microbial structure in HFD-fed ApoE^−/−^ mice. They administered BBR in drinking water (0.5 g/L) to mice for 14 weeks and found that BBR treatment significantly reduced AS in HFD-fed mice, accompanied by a significant increase in the abundance of *Akkermansia* spp. Another study performed in HFD-fed ApoE^−/−^ mice showed that BBR gavage treatment reduced the development of AS and expression of inflammatory cytokines, and the gut microbial profile was significantly different from that without BBR treatment. Specifically, BBR treatment changed the abundance of Firmicutes and Verrucomicrobia, which are two important components of the gut microbiota at the phylum level (Shi et al., 2018). In addition, the abundance of *Alistipes*, *Allobaculum*, *Blautia*, *Roseburia*, and *Turicibacter* was enriched in HFD-fed ApoE^−/−^ mice after treatment with different doses of BBR (Wu et al., 2020). Although these studies indicate that BBR alters the gut microbiota of AS mice, directly linking AS with the effect of BBR on gut microbiota is still required, and further bacterial transplantation experiments may be needed to clarify this.

### Anti-Atherosclerotic Effects of Berberine Through the Regulation of Gut Microbiota-Dependent Metabolites

#### Trimethylamine N-Oxide

TMAO is one of the most important metabolites of the gut microbiota (Wang and Zhao, 2018). Dietary choline, L-carnitine, and betaine are the main nutritional precursors, which are metabolized into trimethylamine (TMA) with the participation of gut microbiota (Wang et al., 2011b; Tang and Hazen, 2014). After being absorbed in the intestine, TMA is delivered to the liver via the portal circulation and is further converted to TMAO by hepatic flavin monooxygenases (FMOs) (Bennett et al., 2013). Data from several representative clinical studies, including prospective cohort studies, suggest that TMAO levels are independently associated with the risk of AS and related cardiovascular diseases (Wang et al., 2011b; Koeth et al., 2013; Stubbs et al., 2016) and can predict both the short- and long-term risk of major adverse cardiovascular events (MACE) (Tang et al., 2013; Tang et al., 2014; Wang et al., 2014; Li et al., 2017b; Li et al., 2019). The potential of TMAO as a therapeutic target for AS and ASCVDs has been explored and strongly supported by animal models and independently replicated clinical studies (Yang et al., 2019a). Current studies have shown that the therapeutic mechanism involves the inhibition of various steps of TMAO metabolism. Examples include dietary choices (Sofi et al., 2008; De Filippis et al., 2016) and lifestyle interventions (Leal-Witt et al., 2018; Erickson et al., 2019); the use of antibiotics, probiotics, and probiotic functional products, as well as some natural molecules that regulate the gut microbiota (Wang et al., 2011b; Tang et al., 2013; Chen et al., 2016; Huang et al., 2018); and inhibition of TMA production and the transformation of TMA to TMAO, thereby reducing plasma TMAO levels. BBR, as a natural Chinese medicinal ingredient, also acts on the above process to regulate the level of TMAO and exhibits an anti-atherosclerotic effect.

The ability of BBR to regulate the composition and structure of the gut microbiota was introduced in the previous chapter. As mentioned above, TMA is the precursor of TMAO. Wang et al. first demonstrated that 3,3-dimethyl-1-butanol (DMB, a choline structural analog), and its modified product could attenuate TMA formation non-lethally by inhibiting microbial TMA lyase (*cutC* and *cutD*), significantly reducing plasma TMAO levels and alleviating atherosclerotic lesions in diet-induced animal models (Wang et al., 2015; Roberts et al., 2018). A recent study published by Li et al. (2021) further confirmed these results, as BBR was found to reduce TMA/TMAO production in choline-fed C57BL/6J and ApoE knockout mice and attenuate atherosclerotic lesion areas in the latter. This was accompanied by alterations in gut microbiota composition and function as well as altered *cutC*/*cntA* gene abundance. Data obtained from *in vitro* and fecal samples of choline-fed mice and human volunteers indicated that BBR inhibited the conversion of choline to TMA, and this conclusion was confirmed via transplantation of TMA-producing bacteria (including *Anaerococcus hydrogenalis, Clostridium asparagiforme, Clostridium sporogenes,* and *Escherichia fergusonii*) in mice (Li et al., 2021). These findings are also similar to those of our previous study on the effect of BBR on AS and gut microbiota regulation in HFD-fed ApoE^−/−^ mice. In our previous work, after the administration of BBR for 13 weeks, metagenomic analysis showed that the abundance of microbial enzymes mainly involved in TMA production, including choline TMA lyase, betaine reductase, and L-carnitine CoA-transferase, was significantly reduced in both the high- (100 mg/kg) and low-dose (50 mg/kg) BBR groups, suggesting a reduced potential for TMAO production (Wu et al., 2020). Flavin containing dimethylaniline monooxygenase 3 (FMO3) is a key enzyme in the conversion of TMA to TMAO. Its silencing or overexpression directly affects systemic TMAO levels and changes cholesterol and lipid metabolism, as well as platelet reactivity and thrombosis in different mouse strains (Bennett et al., 2013; Miao et al., 2015; Schugar and Brown, 2015; Warrier et al., 2015; Zhu et al., 2018b). Shi et al. (2018) cohoused BBR-treated, HFD-fed mice with non-HFD-fed mice and found that both cohousing and BBR treatment significantly decreased liver FMO3 expression and serum TMAO levels, and reduced the development of AS and expression of inflammatory cytokines.

Owing to the complexity of the body, suppressing TMAO production may cause many problems. For example, chronic use of antibiotics to regulate gut microbiota and inhibit TMAO may result in obesity and IR (Cho et al., 2012; Tang and Hazen, 2014). The inhibition of FMO3 expression leads to massive accumulation of TMA and trimethylaminuria, which seriously affects patient quality of life (Treacy et al., 1998). In addition to the systemic role of FMO3 in catecholamine metabolism and other metabolic processes, over-targeted inhibition of its function is probably not harmless (DiNicolantonio et al., 2019). Owing to the above limitations, BBR, a natural Chinese medicinal ingredient, has attracted increasing attention as a promising therapeutic for regulating TMAO levels and alleviating AS. Of note, the regulatory mechanisms of the downstream pathways of TMAO by BBR require further study. Current studies have shown that TMAO may affect the progression of AS by the activation of immune and inflammatory responses, alteration of cholesterol metabolism, and promotion of atherosclerotic thrombosis (Yang et al., 2019a). However, further studies are needed to provide more direct evidence on whether BBR plays an anti-AS role by acting on downstream pathways to regulate the gut microbiota-driven metabolite TMAO.

#### Short-Chain Fatty Acids

SCFAs are one of the most well-studied gut microbiota-derived metabolites, the most abundant of which are acetate, butyrate, and propionate (Brown and Hazen, 2018). Brandsma et al. (2019) transplanted proinflammatory caspase 1 (Casp1)(−/−) microbiota into low density lipoprotein receptor (Ldlr)(−/−) mice using a cohousing method and found that the SCFA-producing taxonomies, *Akkermansia*, *Odoribacter*, *Clostridium*, and *Christensenellaceae* were significantly reduced in the Ldlr(−/−)(Casp1(−/−)) mice. Concordant with these results, the cumulative concentrations of anti-inflammatory SCFAs, including acetate, propionate, and butyrate, were also remarkably reduced, accompanied by increased systemic inflammation and accelerated AS. SCFAs also affect host immune homeostasis. In ApoE^−/−^ mice, propionate treatment significantly reduced aortic atherosclerotic lesion area and systemic inflammation, the latter quantified as decreased splenic effector memory T cell frequency and splenic T helper 17 (Th17) cells (Bartolomaeus et al., 2019). Notably, evidence from both *in vivo* and *in vitro* studies suggests that BBR regulates the production of SCFAs in the gut microbiota and alleviates AS progression. *In vitro* bacterial fermentation experiments showed that BBR increased the concentration of SCFAs in a concentration- and time-dependent manner, which was specifically manifested in significantly upregulated gene expression of key enzymes involved in SCFA synthesis, including acetate kinase (ACK), methylmalonyl-CoA decarboxylase (MMD), and butyryl-CoA: acetate-CoA transferase (BUT) (Wang et al., 2017c). The experiments conducted by Wu et al. (2020) on HFD-induced ApoE^−/−^ AS model mice showed that *Alistipes*, *Allobaculum*, *Blautia*, and *Roseburia*, which were positively correlated with SCFA production, were significantly enriched after high- and low-dose BBR treatments. Consistent with these findings, metagenomic analysis suggested an increased synthesis potential of acetate and butyrate in both BBR groups, which was quantified as an increase in the expression of key enzymes required for synthesis. In particular, the production of acetic acid is significantly related to the relative abundance of *Alistipes* (Yin et al., 2018; Wu et al., 2020)*.* Another study performed in HFD-fed rats indicated that the SCFA-producing genera, *Allobaculum* and *Blautia*, were selectively enriched after BBR treatment, along with increased SCFA concentrations in feces and relief of systemic inflammation (Zhang et al., 2012).

Notably, several studies have revealed that SCFAs potentially prevent or alleviate risk factors of ASCVDs, including obesity, dyslipidemia, diabetes, hypertension, and nonalcoholic fatty liver disease (Hu et al., 2018; Yang et al., 2020). The existing studies listed above have also confirmed that the anti-AS effect of BBR may be partly due to the regulation of gut microbial-derived SCFAs. However, unlike TMAO, few studies have shown a clear association between SCFAs and the risk of ASCVDs in humans (Brown and Hazen, 2018). Therefore, the potential of SCFAs as anti-AS therapeutic targets of BBR requires strong support from additional clinical studies and mechanistic research.

## Berberine Pharmacology and Gut Microbiota in Metabolic Diseases

### Obesity

#### Anti-Obesity Effects of Berberine Through the Alteration of Gut Microbiota

Increasing evidence shows that obesity-mediated changes in the gut microbiota are the key targets of BBR as an anti-obesity agent. Obesity is caused by an imbalance in energy intake, consumption, and storage in the body. Differences in energy acquisition and regulation may be related to the composition of gut microbiota (DiBaise et al., 2008). Experimental evidence from both humans and mouse models showed that obese individuals have a lower relative proportion of Bacteroidetes in their gut microbiota and a correspondingly higher abundance of Firmicutes compared to their lean counterparts (Ley et al., 2005; Ley et al., 2006; DiBaise et al., 2008). In particular, a 50% reduction in Bacteroidetes abundance and a 10-fold increase in the F/B (Firmicutes/Bacteroidetes) ratio were observed in obese mice, which were inhibited by BBR treatment (Sun et al., 2018). This result was also supported by Xie et al. (2011), who showed that BBR (200 mg/kg) administration significantly altered the fecal levels of Firmicutes and Bacteroidetes in HFD-fed mice. In in vitro trials, BBR markedly suppressed the growth of *Lactobacillus*, a typical Firmicutes bacterium, under anaerobic conditions. These results suggest that BBR contributes to the regulation of energy balance in obese individuals by modulating the composition of the gut microbiota. In addition, after 8-week BBR (200 mg/kg) treatment, the reduction in protective bacteria, such as *Bifidobacterium*, in HFD-fed obese rats was significantly reversed. Triglyceride (TG), low-density lipoprotein cholesterol (LDL-C), fasting blood glucose (FBG), and IR were also significantly reduced after BBR treatment, the mechanism of which may be at least partially due to gut microbiota modulation and inhibition of LPS-induced TLR4/TNF-α activation in the liver (Liu et al., 2018).

#### Anti-Obesity Effects of Berberine Through the Regulation of Short-Chain Fatty Acids

The effect of BBR on the inflammatory state and energy homeostasis of the obese body is related to gut microbiota-derived SCFAs. In HFD-induced obese rats, BBR and metformin markedly reduced the diversity of gut microbiota along with a significant enrichment of SCFA-producing bacteria, including *Allobaculum*, *Blautia*, *Bacteroides*, *Butyricicoccus*, and *Phascolarctobacterium* (Zhang et al., 2015)*.* A study by Zhang et al. (2012) further supported these results; these researchers found that HFD-fed rats showed reduced food intake and significantly decreased gut microbiota diversity after BBR treatment. Their findings suggest that the effect of BBR in preventing obesity and IR is due to, at least in part, the regulation of gut microbiota structure, which may be attributed to the alleviation of inflammation by reducing the host's intestinal exogenous antigen load and increasing the levels of SCFAs.

#### Anti-Obesity Effects of Berberine Through Appetite and Energy Metabolism Regulation

BBR decreased food intake, decreased weight gain, and enhanced lipolysis in HFD-fed mice in several studies (Hu and Davies, 2010; Sun et al., 2016). To some extent, such findings may raise questions about the effect of BBR on appetite. Modulation of BBR on the microflora-gut-brain axis was revealed in a study performed in rats fed with HFD, which may be a possible mechanism (Sun et al., 2016). After BBR gavage, structure and diversity alterations in gut microbiota were observed, along with the upregulated expression of glucagon-like peptide-1 (GLP-1) receptor in the brain, elevated serum levels of GLP-1 and neuropeptide Y (NPY), reduced orexin A levels, and improved ultrastructure of the hypothalamus (Sun et al., 2016). In another study, BBR prevented diet-induced obesity by regulating gut microbiota, thereby improving metabolic endotoxemia and aberrant levels of intestinal hormones, such as peptide YY, GLP-1, and GLP-2 (Xu et al., 2017). BBR-induced increases in GLP-1 have been shown to be achieved by enhancing the secretion and biosynthesis of GLP-1 in enteroendocrine L cells and some neurons, which can induce satiety and reduce food intake (Hu and Davies, 2010). This may be one mechanism whereby BBR acts on obese experimental animals and even humans to lower body weight, increase lipolysis, and decrease IR. L-cell dysfunction has been reported as a reason for decreased GLP-1 levels in T2D (Sun et al., 2018). Therefore, to a certain extent, there is crosstalk between the anti-obesity effect and anti-diabetic activity of BBR.

#### Anti-Obesity Effects of Berberine Involving Mitochondrial Energy Homeostasis Regulation

Mitochondrial energy homeostasis is an important metabolic factor associated with obesity. Sun et al. (2018) explored mitochondrial function in diet-induced obese mice. They observed decreased GLP-1 expression in these mice accompanied by mitochondrial stress responses in colonic enterocytes, which are believed to be associated with colon biological dysbiosis and reduced SCFA levels. However, after 8 weeks of BBR treatment (100 mg/kg/d), the mitochondrial stress response was attenuated and GLP-1 expression was restored. The increased expression of fasting-induced adipose factor (Fiaf), an important protein negatively regulated by the gut microbiota, may drive the expression of genes associated with mitochondrial energy metabolism. In C57BL/6J mice fed HFD, BBR (200 mg/kg) significantly increased the expression of Fiaf in intestinal or visceral adipose tissues and enhanced the mRNA expression of mitochondrial energy metabolism-related genes, such as AMP-activated protein kinase (AMPK), carnitine palmitoyltransferase 1α (CPT1α), peroxisome proliferator-activated receptor-γ coactivator 1-α (PGC1α), uncoupling protein 2 (UCP2), and hydroxyacyl-CoA dehydrogenase trifunctional multienzyme complex subunit β (HADHb) (Xie et al., 2011). BBR has also been reported to inhibit mitochondrial complex I in the liver and gut, ameliorate mitochondrial swelling, and facilitate mitochondrial fusion, thus relieving lipid metabolism disorders and alleviating obesity and fatty liver. However, fecal microbiota transplantation indicated that this process was independent of the gut microbiota (Yu et al., 2021). In addition, BBR decreased lipid accumulation in experimental animal adipocytes in a dose- and time-dependent manner through AMPK-dependent and -independent mechanisms (Yang et al., 2019b). This may provide important inspiration and ideas for the prevention and treatment of obesity in humans.

### Diabetes

The antidiabetic effects of BBR have been demonstrated in numerous clinical studies and experimental models. Studies with diabetic patients have shown that BBR has a hypoglycemic effect comparable to that of rosiglitazone and metformin (Yin et al., 2008; Zhang et al., 2010). The mechanism by which BBR lowers glucose levels involves modulating insulin signaling (Liu et al., 2010), acting as an insulin sensitizer and insulinotropic agent (Zhang et al., 2010; Wang et al., 2011a), inhibiting α-glucosidase (Pan et al., 2003), inducing glycolysis, and inhibiting gluconeogenesis (Jiang et al., 2015). In recent years, many studies have found that changes in gut microbiota profiles in adults are associated with T2D, and differences in gut microbiota structure and composition between normal and T2D patients were observed, which may be therapeutic targets for BBR (Larsen et al., 2010; Doumatey et al., 2020).

#### Antidiabetic Effects of Berberine by Regulating Gut Microbiota

Alterations in gut microbiota composition are related to changes in glucose metabolism under pathological diabetic conditions. A study by Pan et al. (2019) conducted in fish showed that BBR may affect serum glucose levels through structural regulation of gut microbiota. The F/B ratio markedly decreased after BBR treatment, and the enriched operational taxonomic units (OTUs) changed from mainly belonging to the dominance of Firmicutes to Proteobacteria, Planctomycetes, Bacteroidetes, and Firmicutes. In rats, BBR fumarate observably ameliorated T2D, increased the abundance of *Alloprevotella*, Bacteroidetes, Clostridia, Lactobacillales and Prevotellaceae, and decreased the abundance of Bacteroidales, *Desulfovibrio*, Lachnospiraceae and Rikenellaceae*.* Reduced inflammation and increased expression of oxidative stress-related proteins (such as phosphoinositide 3-kinases (PI3K) and glucose transporter 2 (GLUT2)) were also observed after treatment, thereby promoting glucose metabolism (Cui et al., 2018). Notably, a recent study in diabetic Goto-Kakizaki rats showed that BBR improved metabolic parameters (including weight, FBG, GLP-1, and homeostatic model assessment-IR); however, the investigators observed significantly lower Bacteroidetes levels and B/F ratio, as well as decreased Muribaculaceae and increased *Allobaculum* levels. Further analysis showed a correlation between certain microbial communities and metabolic parameters (Zhao et al., 2021). As mentioned earlier, there is a crosstalk between the antidiabetic and anti-obesity effects of BBR. Alterations in gut microbiota were characterized by a decrease in protective bacteria, such as *Bifidobacterium*, and an increase in gram-negative bacteria, such as *Escherichia coli*, in HFD-fed obese rats, which led to increased LPS release and TLR4/TNF-α activation. However, BBR treatment (200 mg/kg) reversed these effects, leading to increased expression of the insulin receptor and insulin receptor substrate-1 in the liver, which indicated reduced IR (Liu et al., 2018). Significant reductions in biochemical and morphological markers of diabetes, such as GLP-1 and GLP-2, were also ameliorated after BBR treatment by regulating the microbiota, thereby improving IR and diabetes progression (Shan et al., 2013; Sun et al., 2016). In addition, BBR could slow the progression from prediabetes to T2D in Zucker diabetic fatty rats by increasing intestinal GLP-2 secretion and improving intestinal permeability and gut microbiota structure (Wang et al., 2021a). These findings suggest that regulating the gut microbiota composition to exert hypoglycemic effects is an effective therapeutic use of BBR.

#### Antidiabetic Effects of Berberine by Regulating Gut Microbiota-Dependent Metabolites

The regulation of gut microbiota metabolites may be one of the pivotal mechanisms of BBR in regulating energy metabolism and exerting hypoglycemic effects. A study by Wang et al. (2017d) revealed that the oral administration of BBR in animals facilitated the production of butyrate (an SCFA) by gut microbiota, thereby reducing blood glucose and lipid levels. The mechanism might involve BBR enriching butyrate-producing bacteria and inhibiting bacterial ATP production and the level of NADH, which eventually led to the upregulation of enzymes related to butyrate production in bacteria, thereby promoting butyrate production. Similarly, a recent study reported that Gegen Qinlian decoction (GQD), a traditional Chinese medicine (TCM) formulation, had similar effects to BBR. Both treatments significantly changed the overall structure of the gut microbiota in a diabetic rat model and enriched butyrate-producing bacteria, such as *Faecalibacterium* and *Roseburia*, accompanied by significantly elevated SCFA levels in rat feces. This alleviated intestinal inflammation and lowered blood glucose (Xu et al., 2020). In addition, BBR and metformin have similar hypoglycemic effects. Both treatments effectively increased the abundance of SCFA-producing bacteria, restored the intestinal SCFA content, and reduced blood glucose levels and diabetic complications in a db/db mouse model (Zhang et al., 2019). In an HFD-fed rat model, selective enrichment of SCFA-producing bacteria and increased concentrations of SCFA in the feces were observed after BBR treatment, which may contribute to the alleviation of inflammation, thereby preventing obesity and IR (Zhang et al., 2012).

In addition to SCFAs, elevated circulating branched-chain amino acids (BCAAs) are involved in the pathogenesis of obesity and IR. *In vivo* and *in vitro* experiments showed that the improvement in IR by BBR was associated with a decrease in the relative levels of BCAA-producing bacteria (e.g., Clostridiales, Streptococcaceae, Clostridiaceae, Prevotellaceae, *Streptococcus*, and *Prevotella*) as well as an increase in BCAA catabolism in the liver and adipocytes (Yue et al., 2019). In T2D rats, BBR increased the richness and diversity of the gut microbiota and noticeably decreased the concentration of aromatic amino acids (AAAs) in the colon and serum, including tyrosine, tryptophan, and phenylalanine, thereby alleviating symptoms (Yao et al., 2020). Further studies on the amino acid metabolites of gut microbiota may be needed to identify more specific mechanistic pathways.

In addition, the gut microbiota mediates the metabolism of bile acids (BAs). The BA response receptor involved in host metabolism in this process is Takeda G protein-coupled receptor 5 (TGR5), which is mainly activated by the secondary BAs, lithocholic acid (LCA) and deoxycholic acid (DCA) (Pathak et al., 2018). A study by Li et al. (2020c) with db/db mice showed that BBR compounds increased the relative abundance of Bacteroidaceae and Clostridiaceae, which may have promoted the transformation of the primary BA, cholic acid (CA), to the secondary BA, DCA. Increased microbiota-mediated DCA production upregulated the expression of the bile acid receptor, TGR5, and induced the secretion of GLP, thereby improving hyperglycemia. However, another study of 499 newly diagnosed T2D patients in China suggested that the hypoglycemic effect of BBR was mediated by the suppression of DCA biotransformation by *Ruminococcus bromii* (Zhang et al., 2020).

#### Antidiabetic Effects of Berberine Combined With Probiotic Biotherapy

The use of probiotic biotherapy to maintain proper gut microbiota may be an effective early interventional approach for hyperglycemia. A multicenter, double-blind, randomized, parallel-controlled study involving 300 patients investigated the hypoglycemic efficacy and safety of *Bifidobacterium* and BBR administration in newly diagnosed prediabetic or diabetic patients. The primary outcome was the absolute value of fasting glucose; however, the results have yet to be reported (Ming et al., 2018). In animal studies, the combination of BBR and stachyose, a prebiotic substance, has been shown to have better effects on glucose metabolism and intestinal homeostasis than BBR alone by regulating intestinal microbiota (increasing the abundance of *Akkermansia muciniphila*) and fecal metabolomics (Cao et al., 2020; Li et al., 2020d). These findings indicate that BBR combined with probiotics and prebiotics may be a novel therapeutic strategy for the treatment of T2D. In addition, BBR has been shown to be a potentially promising prebiotic that indirectly promotes the growth of *A. muciniphila* in mice by stimulating intestinal mucin secretion (Dong et al., 2021). In human studies, supplementation with *A. muciniphila* improved insulin sensitivity, reduced insulinemia, and decreased plasma levels of total cholesterol and inflammatory markers (Depommier et al., 2019). In addition, a specific membrane protein isolated from *A. muciniphila* improved the gut barrier and metabolism in obese and diabetic mice (Plovier et al., 2017). However, studies on the combination of *Akkermansia* and BBR to improve diabetes have not yet been reported.

### Dyslipidemia

Although the bioavailability of BBR by oral or intragastric administration is low, it has significant lipid-lowering effects (Li et al., 2015; Ju et al., 2018), suggesting that the intestinal tract is a potential effective target for the hypolipidemic effect of BBR. Studies conducted in hyperlipidemic rats and hamster models induced by HFD indicated that BBR treatment had an anti-hypercholesterolemia effect and modulated the gut microbiota, leading to the enrichment of beneficial bacteria (such as *Bacteroides* and *Brucella*), reduction in *E. coli*, and increase in the F/B ratio (Gu et al., 2015; Li et al., 2016).

The lipid-lowering effect of BBR by modulating the gut microbiota is accompanied by the regulation of gut microbiota-derived metabolites, including SCFAs. Two weeks of treatment with BBR (oral, 200 mg/kg) significantly increased the level of blood butyrate in hyperlipidemic hamsters, and BBR metabolites, such as M1, M2, and M3, significantly induced the production of SCFAs in the gut microbiota (Ma et al., 2019). Another similar animal study showed that BBR (administered orally) increased the abundance of butyrate-producing bacteria and promoted the production of butyrate by the gut microbiota, which then entered the blood and lowered blood lipid levels (Wang et al., 2017d). Notably, intraperitoneal administration of BBR did not increase butyrate but decreased blood lipid levels, indicating that the lipid-lowering effect of BBR may include two action modes: a direct effect of circulating BBR and indirect effect of butyrate through the gut microbiota (Wang et al., 2017d).

BAs are another class of metabolite. Primary BAs, including CA and chenodeoxycholic acid (CDCA), are generated from cholesterol in the liver via the classical pathway catalyzed by CYP7A1 and alternative pathway initiated by CYP27A1, and are then combined with glycine and taurine to form conjugated BAs, which are released into the intestine. Among them, 95% of conjugated BAs are reabsorbed in the ileum and returned to the liver through enterohepatic circulation, while the remainder are deconjugated by bacteria via bile acid hydrolase (BSH) activity and then catalyzed to form secondary BAs, including DCA and LCA (Wahlström et al., 2016). The lipid-lowering effect of BBR is related to the regulation of bile acid metabolism and the bile acid receptor (also known as farnesoid X receptor (FXR)) signaling pathway. Both are closely related to the gut microbiota. For example, metagenomic analyses revealed the taxonomic and abundance profiling of BSH in human gut microbiota (Song et al., 2019) and functional BSH exists in all major bacterial classifications of the human gut, including *Clostridium*, *Bacteroides*, and *Lactobacilli* (Jones et al., 2008; Wahlström et al., 2016). In experimental animals, BBR exposure directly altered gut microbiota by decreasing *Clostridium* cluster XIVa and IV as well as BSH activity, leading to the accumulation of conjugated BAs, especially taurocholic acid (TCA) in the intestine, which promotes intestinal FXR activation (Tian et al., 2019). Further studies showed that BBR and FXR activation result in suppressed hepatic expression of CD36, which leads to decreased uptake of long-chain fatty acids in the liver (Sun et al., 2017). Activation of FXR inhibited the expression of the CYP7A1 gene to regulate BA metabolism and mediate physiological processes, including lipid and glucose metabolism (Goodwin et al., 2000; Sinal et al., 2000). After high-dose BBR administration, *Bacteroides* enrichment in the terminal ileum and large bowel of mice was observed, as well as an increased expression of the BA-synthetic enzymes, CYP7A1 and CYP8B1, and sodium/taurocholate cotransporting polypeptide (NTCP) in the liver, which suggested upregulated BA production (Guo et al., 2016). A study in hyperlipidemic hamster models showed that BBR-regulated molecules related to lipid metabolism increased BA generation, modulated gut microbiota, and exhibited a significant inhibitory effect on the transformation of CA into DCA, which indicated a decreased elimination of intestinal BAs (Gu et al., 2015). A colonization experiment with a minimal intestinal bacterial consortium that is capable of functional BA metabolism in germ-free mice also supported this result. These investigators found that the lipid (particularly cholesterol)-lowering effect of BBR was achieved partly by upregulating cecal BA concentrations and excretion into the gastrointestinal tract (Wolf et al., 2021). These efficacies were also considered to depend on modulations in the structure and function of the gut microbiota.

### Nonalcoholic Fatty Liver Disease

NAFLD is one of the most frequent diseases associated with obesity, T2D, and dyslipidemia and is strictly linked to cardiovascular disease via AS, although the underlying mechanisms are far from understood (Tarantino et al., 2019). Many animal studies have demonstrated that the gut microbiota plays an important role in the development and treatment of NAFLD (Table 1).

One of the likely mechanisms occurs through the improvement of intestinal barrier function. Li et al. conducted experiments in NAFLD rats and found that BBR (150 mg/kg/d for 4 weeks) administration significantly ameliorated HFD-induced hepatic fatty degeneration by increasing occludin levels and improving intestinal barrier dysfunction, accompanied by decreased levels of *Faecalibacterium prausnitzii* and increased levels of *Bacteroides* (Li et al., 2017a). Studies by other groups also indicated that BBR could efficiently protect the gut barrier function of NAFLD rats, and the mechanism may have involved regulation of the gut microbiota, including an increase in *Bacteroidetes* levels and a decrease in *E. coli* and *F. prausnitzii* levels (Li et al., 2020e). Regarding other possible mechanisms for ameliorating NAFLD, recent findings suggest that BBR can reverse the HFD-induced suppression of fatty acid mitochondrial β-oxidation by activating sirtuin 3 (SIRT3)-mediated long-chain acyl-CoA dehydrogenase (LCAD) deacetylation, thereby improving systematic and hepatic lipid metabolism in mice (Xu et al., 2019). Concordantly, a parallel, open-labeled, randomized controlled trial that was performed at three medical centers also suggested that the therapeutic effect of BBR in patients with NAFLD might be related to the regulation of hepatic lipid metabolism. This trial demonstrated that BBR combined with lifestyle treatment significantly reduced hepatic fat content and improved body weight, lipid profiles, and homeostatic model assessment-IR (Yan et al., 2015). Animal experiments have shown that BBR alters the expression of genes related to hepatic metabolism (Yan et al., 2015). In addition to reducing hepatic lipid accumulation by regulating the synthesis and catabolism of fatty acids, BBR restored bile acid homeostasis, which was closely related to gut microbiota as mentioned above, inhibited inflammation, and reduced hepatic fibrosis in a mouse model of NAFLD, thus preventing disease progression (Wang et al., 2021b). These beneficial effects of BBR are related to the downregulation of long non-coding RNA H19 and microRNA 34a (Wang et al., 2021b). In addition, the use of prebiotics and probiotics in the treatment and prevention of obesity-related NAFLD patients has been proposed, but their therapeutic application has not been supported by high-quality clinical research (Tarantino and Finelli, 2015). BBR combined with prebiotics and probiotics in the treatment of NAFLD may be a promising direction worthy of further exploration.

In summary, these findings may provide new insights that support BBR as a promising treatment for NAFLD through the regulation of gut microbiota.

## Conclusion

**FIGURE 2:**
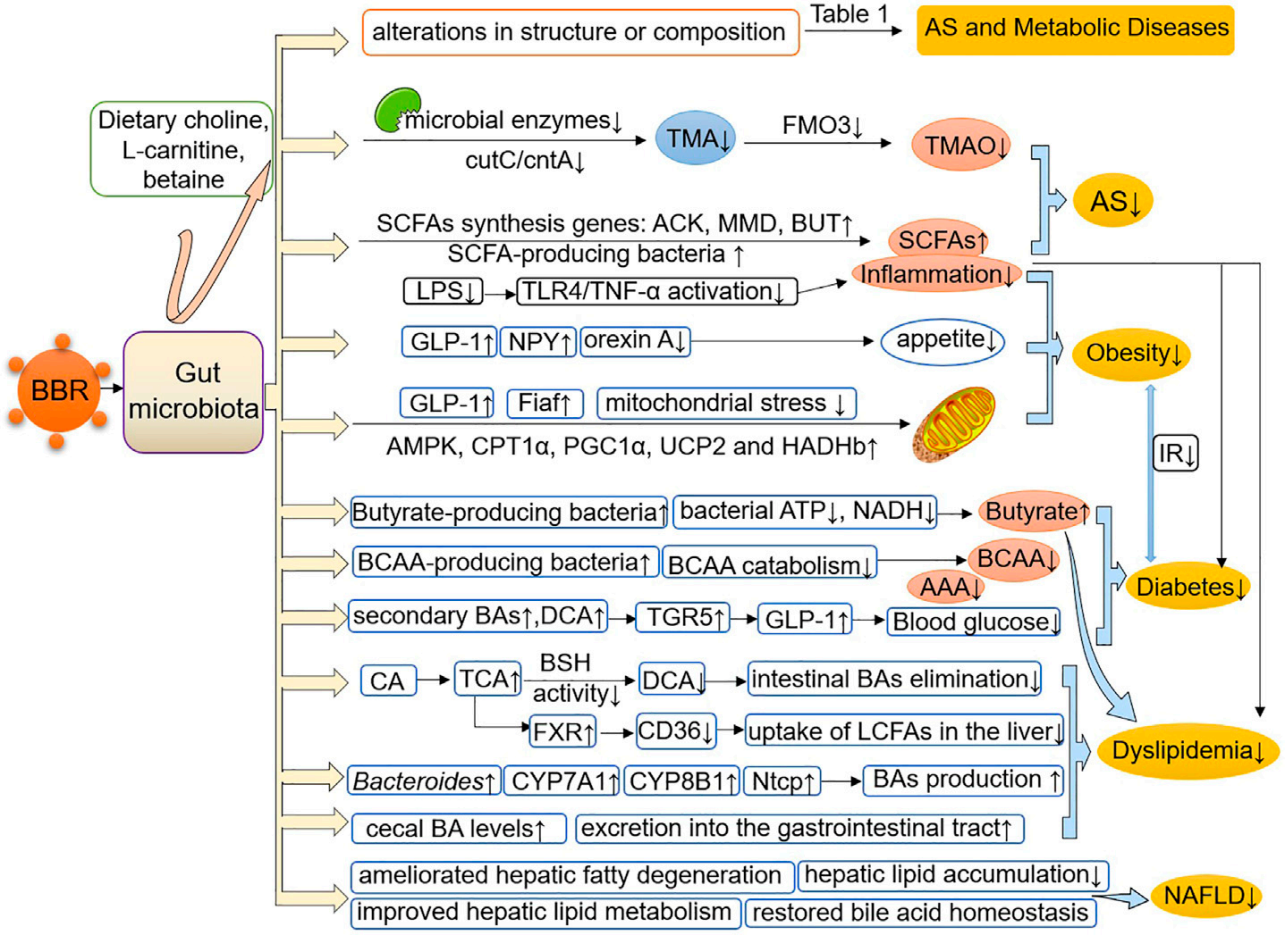
BBR improves AS and metabolic diseases by modulating the gut microbiota and its-dependent metabolites. BBR reverses or induces structural and compositional alterations in the gut microbiota and regulates gut microbe-dependent metabolites, including TMAO, SCFAs, BAs, BCAAs and AAAs, as well as related downstream pathways, thereby improving the body’s inflammatory state, glucose and lipid metabolism and energy homeostasis, and playing a beneficial role in AS and metabolic diseases. AS, atherosclerosis; TMA, trimethylamine; TMAO, trimethylamine N-oxide; ACK, acetate kinase; MMD, methylmalonyl-CoA decarboxylase; BUT, butyryl-CoA: acetate-CoA transferase; SCFAs, short-chain fatty acids; LPS, lipopolysaccharide; TNF-α, tumor necrosis factor-α; GLP-1, glucagon-like peptide-1; NPY, neuropeptide Y; Fiaf, fasting-induced adipose factor; AMPK, AMP-activated protein kinase; CPT1α, carnitine palmitoyltransferase 1α; PGC1α, peroxisome proliferator-activated receptor-γ coactivator 1-α; HADHb, hydroxyacyl-CoA dehydrogenase trifunctional multienzyme complex subunit β; UCP2, uncoupling protein 2; BCAAs, branched-chain amino acids; BAs, bile acids; AAAs, aromatic amino acids; TGR5, Takeda G protein-coupled receptor 5; CA, cholic acid; TCA, taurocholic acid; DCA, deoxycholic acid; BSH, bile acid hydrolase; FXR, farnesoid X receptor; LCFAs, long-chain fatty acids; NTCP, sodium/taurocholate cotransporting polypeptide; NAFLD, nonalcoholic fatty liver disease.

It is a promising domain to explore the multiple pharmacological effects of BBR in AS and metabolic diseases from the perspective of gut microbiota. The research evidence scrutinized herein shows that BBR reverses alterations in the structure, quantity, and composition of gut microbiota under the pathological conditions of AS and metabolic diseases, and induces changes in certain bacterial taxa (Table 1). In addition, BBR improves intestinal barrier function and reduces inflammation in AS and metabolic diseases (Figure 1). Under the pathological conditions of AS and metabolic diseases, increased intestinal permeability has been shown to occur. This promotes systemic inflammation induced by gut microbiota-derived LPS, leading to enhanced IR, IGT, obesity, endothelial dysfunction, and atherosclerotic plaque vulnerability, all of which are ameliorated after BBR treatment. In addition, BBR improves the body’s inflammatory state, glucose and lipid metabolism, and energy homeostasis by regulating gut microbe-dependent metabolites (such as TMAO, SCFAs, BAs, and BCAAs) as well as related downstream pathways, thereby playing a beneficial role in AS and metabolic diseases (Figure 2). It is worth noting that there are still some limitations to existing studies. Although these studies have indicated that BBR alters the gut microbiota of model animals and patients, a direct association between the effect of BBR on gut microbiota and AS and metabolic diseases has not been demonstrated clearly. Moreover, further bacterial transplantation experiments may be needed in the future to provide more direct evidence. More high-quality studies are also needed to explore specific downstream pathways based on the gut microbiota and its metabolites, to better reveal the mechanism of the effect of BBR on AS and metabolic diseases.
